# Supplementary material for: Post-supereruption recovery at Toba Caldera
Source: Nat Commun. 2017 May 16;8:15248. doi: 10.1038/ncomms15248 (PMC5440807; doi:10.1038/ncomms15248)
Supplement: Supplementary Information — Supplementary Figures and Supplementary References [file ncomms15248-s1.pdf]

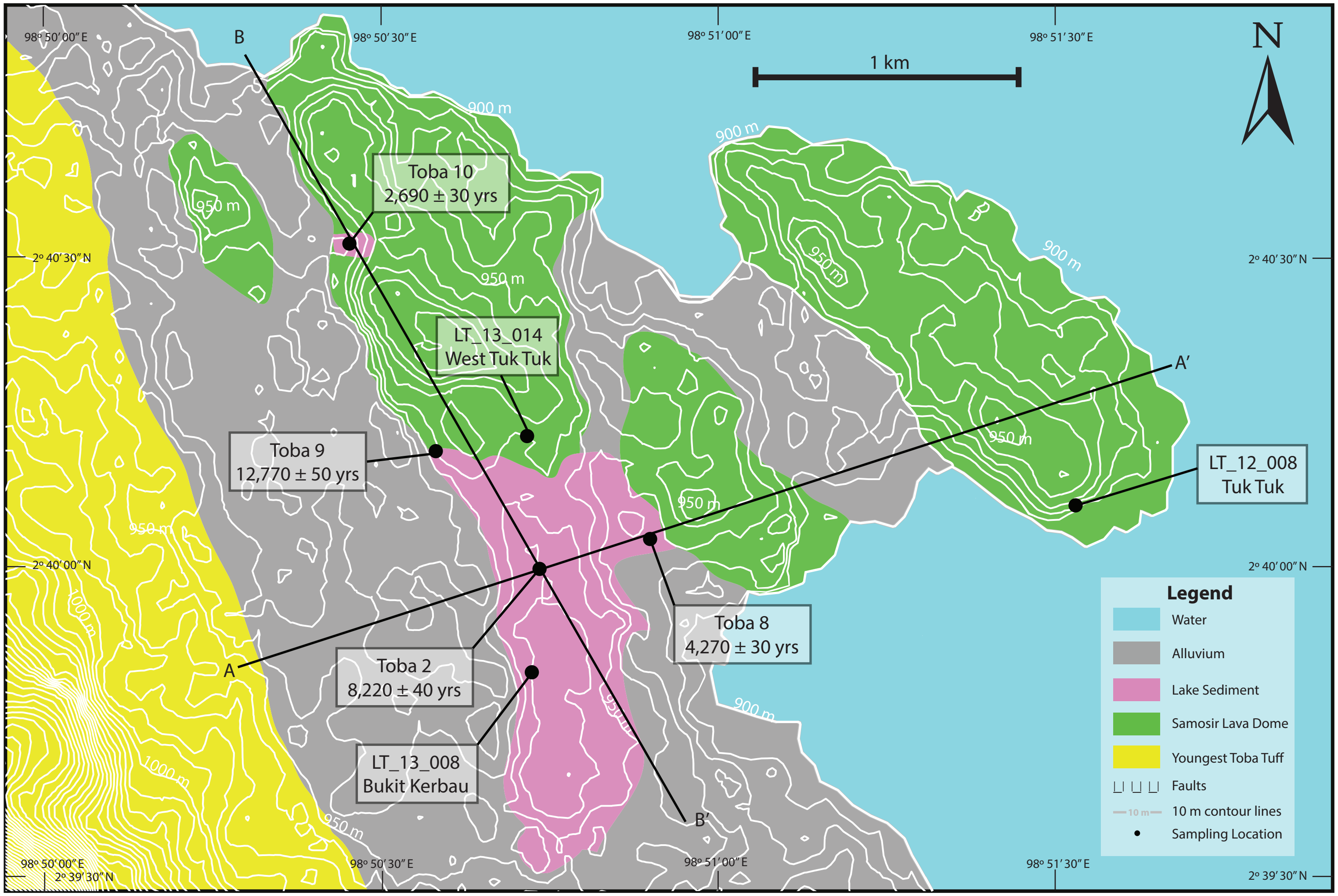

Supplementary Figure 1: Overview location map of lake sediments on Tuk Tuk Peninsula

The Tuk Tuk peninsula is made up of several lobes of lava domes that have faulted to form three sections (Tuk Tuk, West Tuk Tuk, Bukit Kerbau). The lake sediments deposited on top of these lava domes were sampled to constrain the minimum age for the emplacement of the Tuk Tuk lava dome. Sampling locations are indicated by black dots; sediment samples are labeled as found in Supplementary Data 2; lava dome samples are labeled as found in Table 1. Faults are inferred from the surrounding geology; any evidence of fault scarps have either been destroyed through construction or are obstructed by forests growing along the edge of the lava domes.

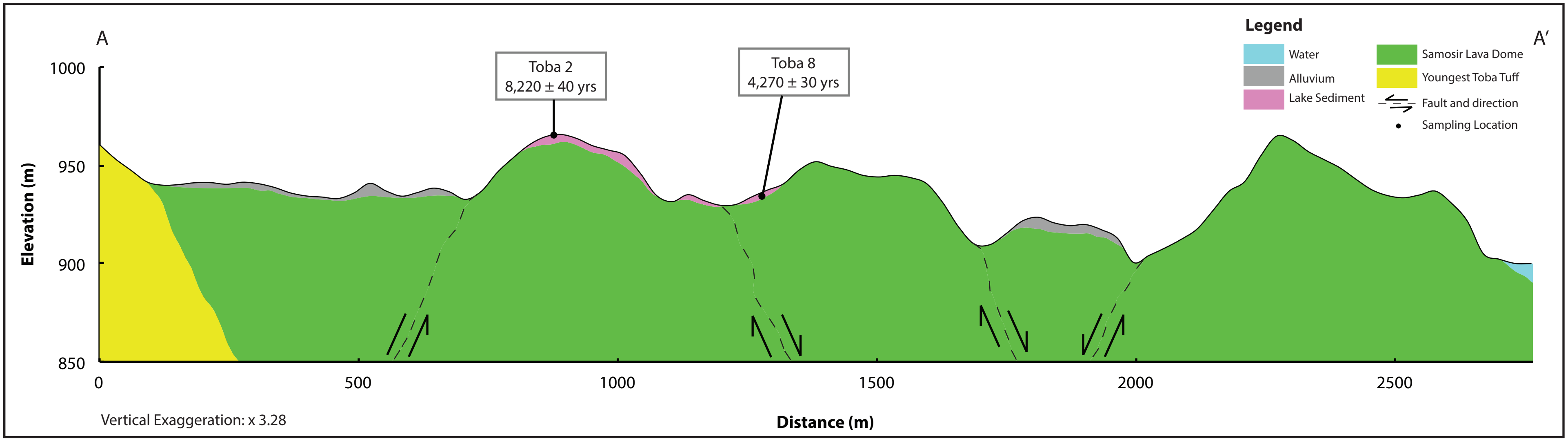

Supplementary Figure 2: A-A’ cross-section across Tuk Tuk peninsula

(A-A’) East-west cross-section of the Tuk Tuk peninsula showing the geology, faulting, and sediment locations from the Samosir fault through the Youngest Toba Tuff to the Tuk Tuk coast (lake level at 900 m).

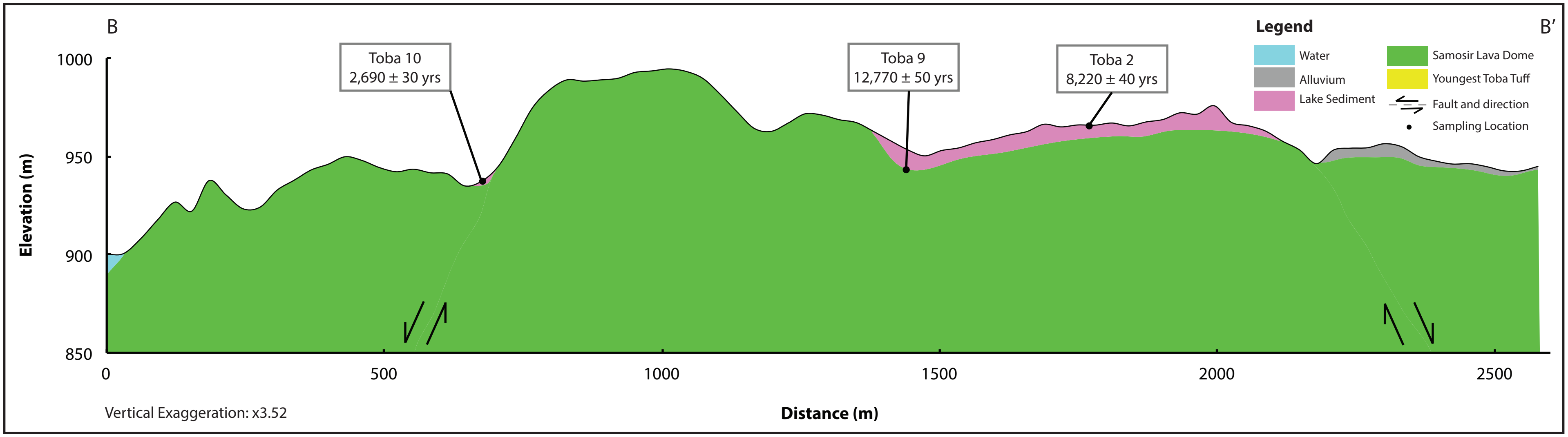

Supplementary Figure 3: B-B' cross-section across Tuk Tuk peninsula

(B-B') North-south cross-section of the Tuk Tuk peninsula showing the geology, faulting and sediment locations along the length of the lava dome, starting at the northern Tuk Tuk coast (lake level at 900 m).

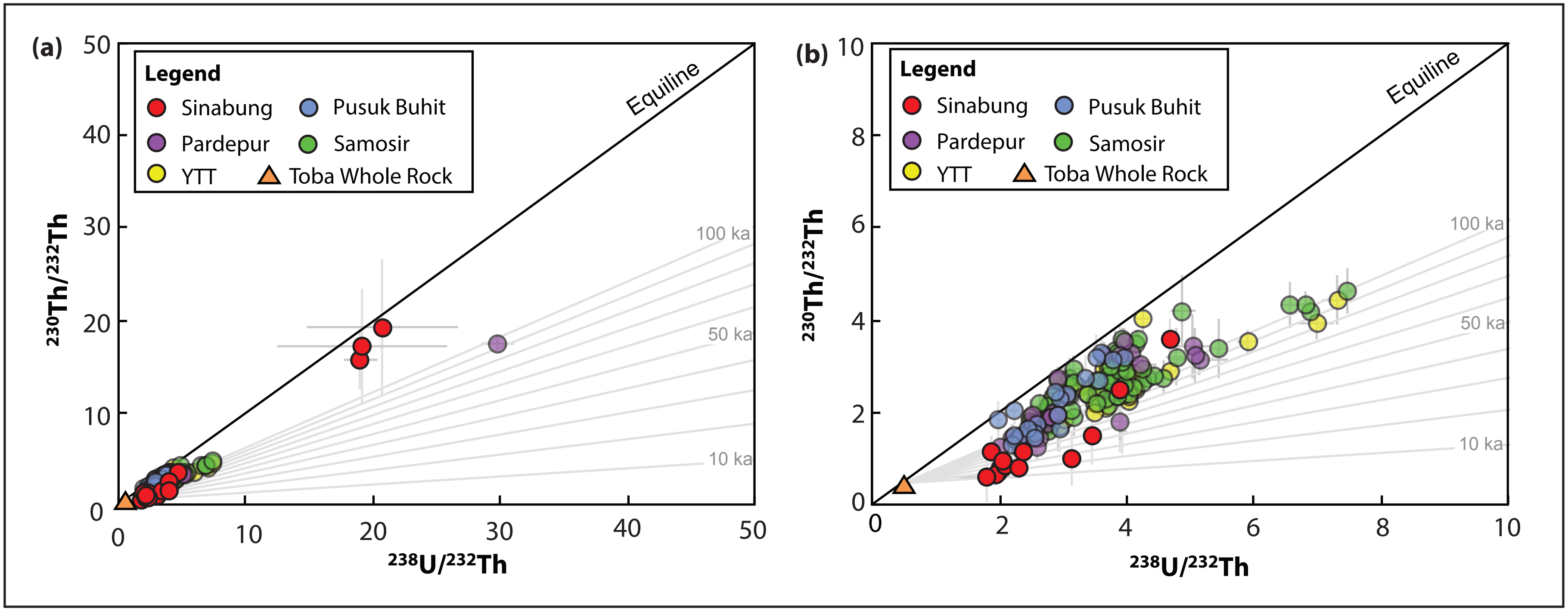

Supplementary Figure 4: ^{238}U-^{230}Th and ^{232}Th-^{230}Th disequilibrium plot of Toba and Sinabung samples

^{238}U-^{230}Th and ^{232}Th-^{230}Th disequilibrium plot of Youngest Toba Tuff, Samosir lava domes, Pardepur lava domes, and Pusuk Buhit lava flows, along with ^{238}U-^{230}Th and ^{232}Th-^{230}Th data from Sinabung. Toba whole rock isotopic compositions are (^{232}Th-^{230}Th) = 0.465 ± 0.004 and (^{238}U-^{230}Th) = 0.517 ± 0.002 (2 standard error (σ))[1]. Faint grey lines are calculated isochrons in 10 kyr increments, spanning the ages of the surface peak ages from 10 ka to 100 ka. Panel (a) shows entire data set, while panel (b) is a close up, showing the data plotted between values of 0 – 10 for both (^{238}U-^{230}Th) and (^{232}Th-^{230}Th). Error bars are ± 2σ; plotted data is found in Supplementary Data 4.

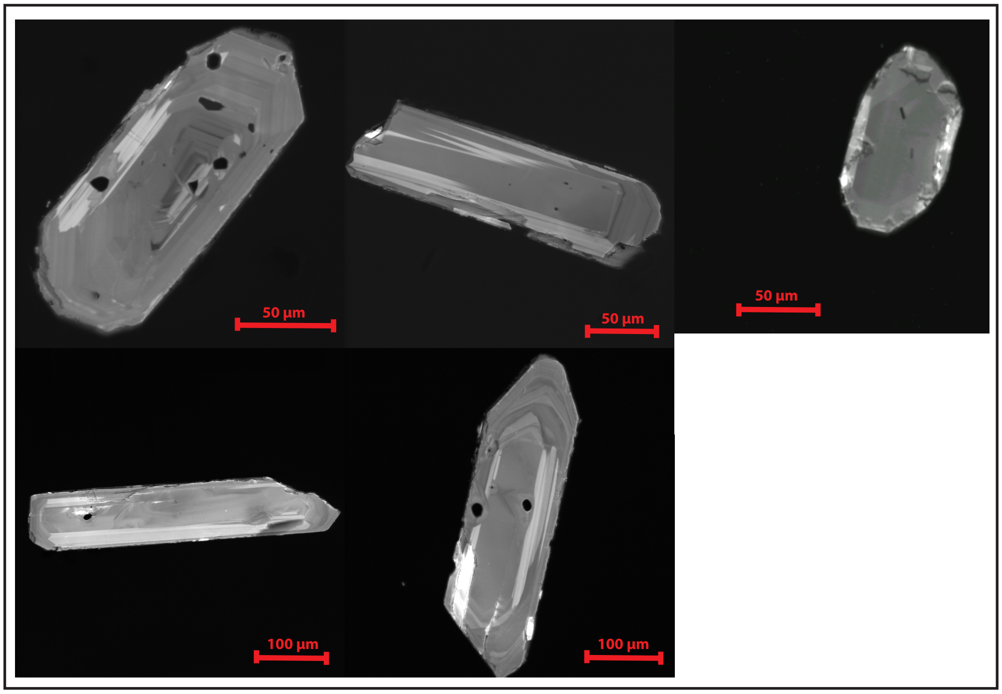

Supplementary Figure 5: Cathodeluminescence (CL) images of representative zircon crystals from Toba and Sinabung samples
Top row (left to right): YTT zircon (LT_12_001), Samosir lava dome zircon (LT_12_003), Sinabung zircon (LT_14_012).
Bottom row (left to right): Pardepur lava dome zircon (LT_12_016) and Pusuk Buhit lava flow zircon (LT_14_007).
Darker regions are relatively enriched in uranium, whereas lighter regions are poor in uranium.

Supplementary References

1. Vazquez, J. A. & Reid, M. R. Probing the accumulation history of the voluminous Toba magma. *Science* **305**, 991-994 (2004).

2. Whitford, D. J. & Jezek, P. A. Geochemistry of Cenozoic and Recent lavas from the Banda arc, Indonesia. *Carnegie I. Wash.* **76**, 845-855 (1977).

3. Chesner, C. A. *The Toba Tuffs and Caldera Complex, Sumatra, Indonesia: Insights into magma bodies and eruptions* Ph.D. thesis, Michigan Tech. Univ. (1988).

4. Jones, S. *Mechanisms of Large Silicic Magma Chamber Zonation: the Youngest Toba Tuff, Sumatra* Ph.D. thesis, The Open Univ. (1989).

5. Gasparon, M. & Varne, R. Sumatran granitoids and their relationship to Southeast Asian terranes. *Tectonophysics* **251**, 277-299 (1995).

6. Alloway, B. V. *et al*. Correspondence between glass-FT and 14 C ages of silicic pyroclastic flow deposits sourced from Maninjau caldera, west-central Sumatra. *Earth Planet. Sc. Lett.* **227**, 121-133 (2004).

7. Sarbas, B. *The GEOROC database as part of a growing geoinformatics network*. In S.R. Brady, A.K. Sinha, and L. C. Gundersen, Eds., Geoinformatics 2008—Data to Knowledge, Proceedings, U.S. Geological Survey Scientific Investigations Report 2008-5172, 42–43 (2008).
